# Supplementary material for: Resensitizing carbapenem- and colistin-resistant bacteria to antibiotics using auranofin
Source: Nat Commun. 2020 Oct 16;11:5263. doi: 10.1038/s41467-020-18939-y (PMC7568570; doi:10.1038/s41467-020-18939-y)
Supplement: Supplementary file 1 — Supplementary Information [file 41467_2020_18939_MOESM1_ESM.pdf]

Supplementary Information

Resensitizing carbapenem- and colistin-resistant bacteria to antibiotics using auranofin

Hongzhe Sun[1†*], Qi Zhang[1†], Runming Wang[1†*], Haibo Wang[1], Yuen-Ting Wong[1,2], Minji Wang[3], Quan Hao[4], Aixin Yan[3], Richard Yi-Tsun Kao[2,5,6], Pak-Leung Ho[2,5,6], and Hongyan Li[1]

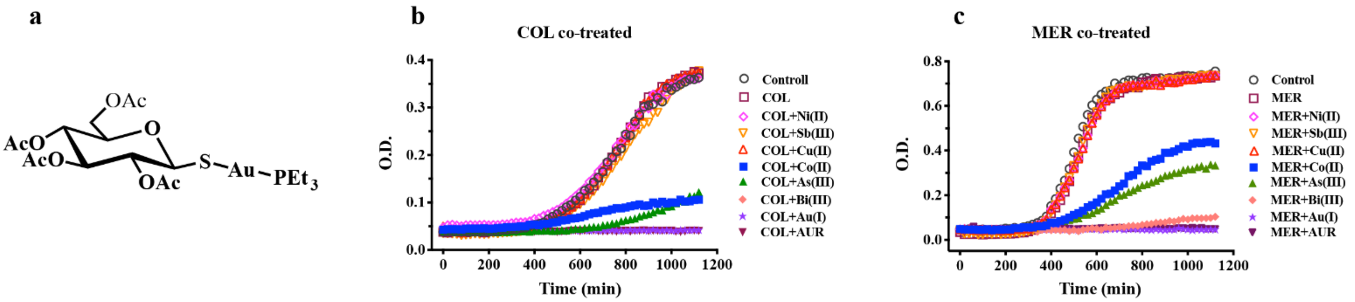

Supplementary Fig. 1 | a, Structure of AUR. **b,c,** Bacterial growth curves show the antimicrobial effect of **(b)** COL and **(c)** MER against *E. coli* CKE (MCR-1^+, NDM-5^+) in the presence of different metal compounds. The concentrations of all metal ions were fixed at 50 μg·mL^{-1} while COL (1 μg·mL^{-1}) and MER (2 μg·mL^{-1}) were used at sub-inhibitory concentration.

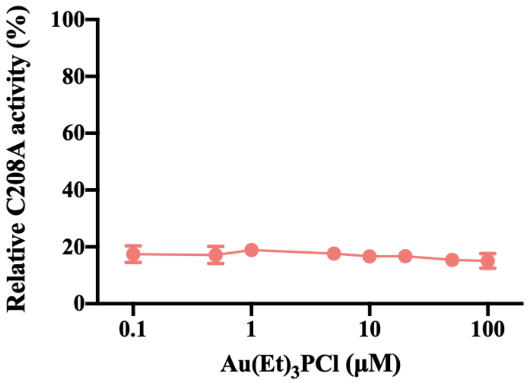

Supplementary Fig. 2 | Inhibition of NDM-1-C208A by Au(PEt$_3$)Cl. Note the low activity of the enzyme and limited inhibition by Au(PEt$_3$)Cl. Mean values of three replicates are shown and error bars indicate ±SEM, n=3 biologically independent samples. Source data are provided with this paper.

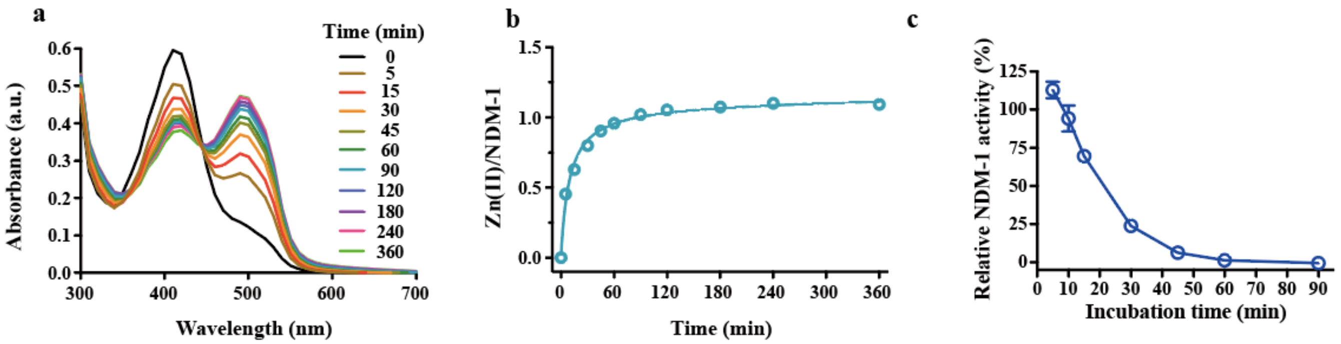

Supplementary Fig. 3 | a, UV-vis spectra of Zn_2-NDM-1 in the presence of Au(PEt_3)Cl as well as PAR reagent at different time points. **b,** Kinetics of Zn(II) released from Zn_2-NDM-1 ($t_{1/2}$ =13.78 min) at different time points. Zn_2-NDM-1 (10 μM) supplemented with 150 μM PAR was titrated with 200 μM Au(PEt_3)Cl at 25 °C and UV spectra was recorded at various time intervals after the addition of Au(PEt_3)Cl. The concentration of released Zn(II) was determined by comparing the absorbance at 490 nm to a Zn(II) standard curve. **c**, Inhibition of activity of NDM-1 that was preincubated with Au(PEt_3)Cl (5 μM) for different time. Data are presented as mean values ±SEM, n=3 biologically independent samples. Source data are provided with this paper.

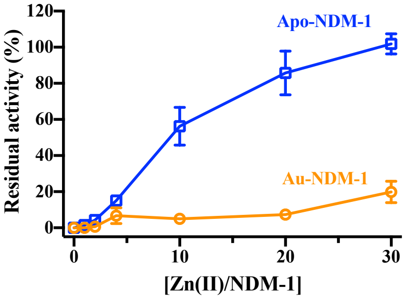

Supplementary Fig. 4 | Restoration of the activity of NDM-1 upon supplementation of various ratios of Zn(II) to apo-NDM-1 and Au-NDM-1, respectively. Mean values of three replicates are shown and error bars indicate ±SEM, n=3 biologically independent samples. Source data are provided with this paper.

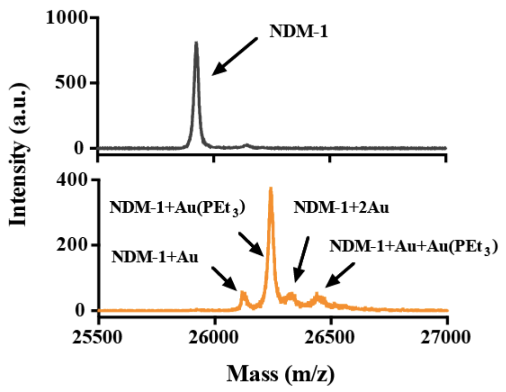

Supplementary Fig. 5 | MALDI-TOF-MS of NDM-1 after incubation with 10 molar equivalence of AUR. The peak at m/z of 25925.4 corresponds to NDM-1 monomer. The peaks at m/z of 26124.3, 26240.7, 26323.1 and 26442.9 were assigned to [NDM-1+Au] (calcd: 26122.4), [NDM-1+Au(PEt$_3$)] (calcd: 26240.5), [NDM-1+2Au)] (calcd: 26319.3), and [NDM-1+Au+Au(PEt$_3$)] (calcd: 26437.5), respectively.

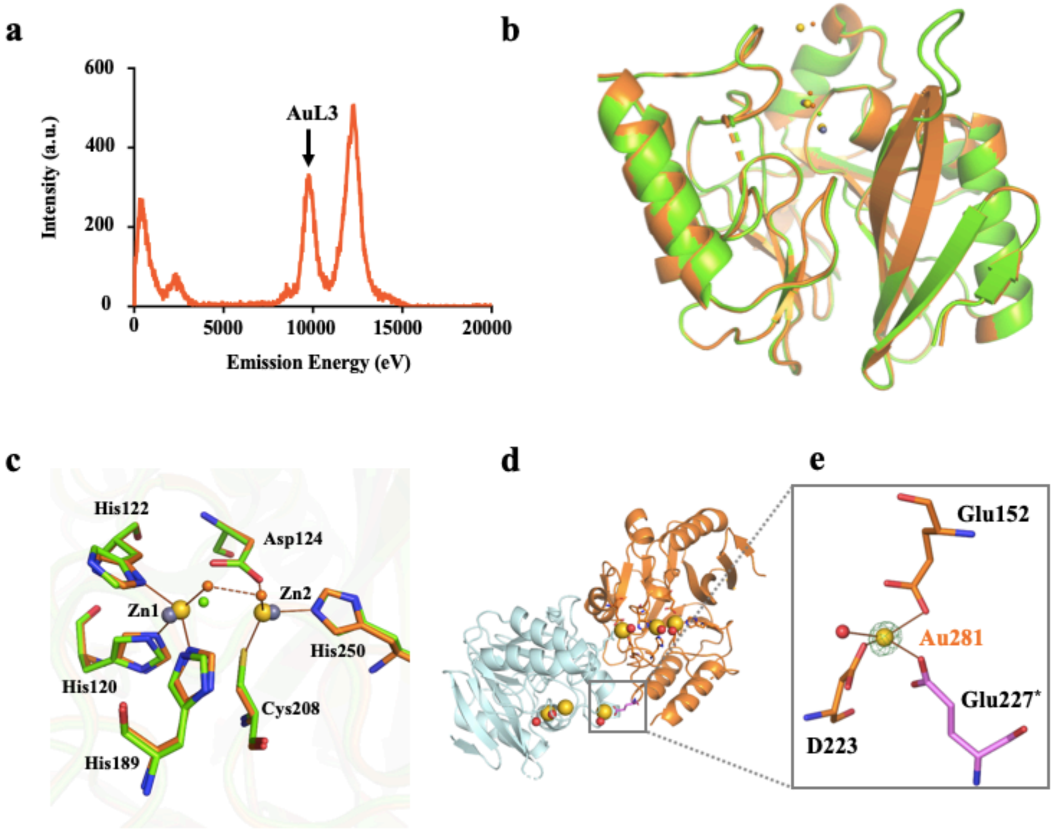

Supplementary Fig. 6 | a, X-ray excitation scans at 0.998 Å for Au-NDM-1 crystal. Gold exhibits emission at 9.8 keV, corresponding to its L3 absorption edge. The emission at around 13 keV is from the background. **b,c,** Superimposition of (**b**) overall structures and **(c)** active site of Au-NDM-1 (PDB ID: 6LHE) (orange) with native Zn-NDM-1(PDB ID: 5ZGE) (green). Au(I) ions are shown as yellow spheres and Zn(II) ions as grey spheres. Structural alignment was done over C_{α} residues using DaliLite and the images were generated using PyMOL. The two structures can be superimposed well with a RMSD value of 0.211 Å. **d,** overall structure of Au-NDM-1. **e**, Binding of Au^{281} to a non-active site of Au-NDM-1. Note that Au^{281} coordinates Asp223, Glu152 as well as a Glu227 from an adjacent symmetric unit.

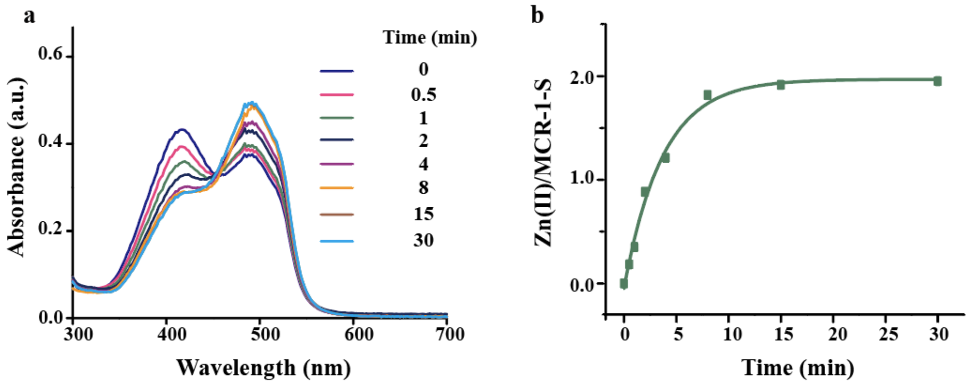

Supplementary Fig. 7 | a, UV-vis spectra of MCR-1-S in the presence of Au(PEt_3)Cl and PAR at different times. **b,** Kinetics of Zn(II) release from Zn_3-MCR-1-S ($t_{1/2}$= 2.54 min). Zn_3-MCR-1-S (20 μM) supplemented with 150 μM PAR was titrated with Au(PEt_3)Cl (200 μM) at 25 °C. The UV spectra was recorded at various time points after the addition of Au(PEt_3)Cl. The concentration of metal released was determined by comparing the absorbance at 490 nm to a Zn(II) standard curve.

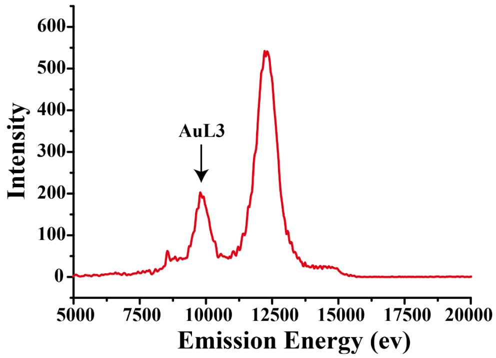

Supplementary Fig. 8 | X-ray excitation scans at 0.998 Å for Au-MCR-1-S crystal. Emission at 9.8 keV was observed, corresponding to L3 absorption edge of gold. The emission at around 13 keV is from the background.

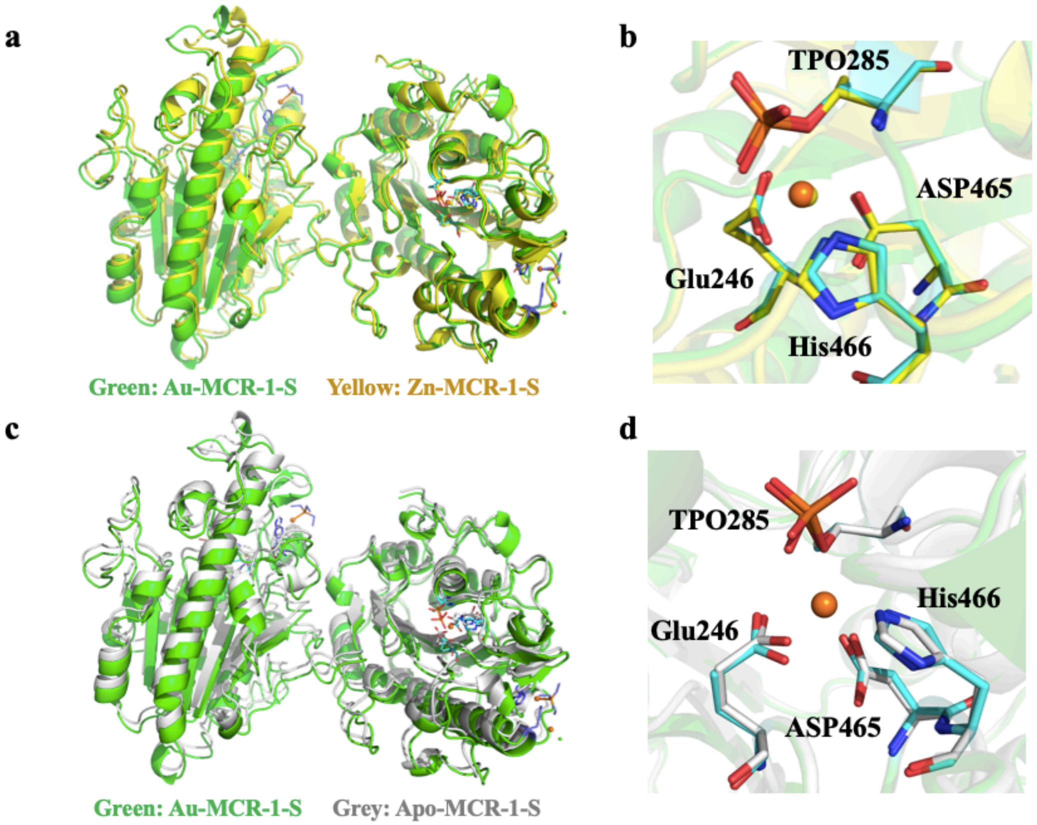

Supplementary Fig. 9 | a,b, Superimposition of **(a)** overall structures and **(b)** active sites of Au-MCR-1-S (PDB ID: 6LI6) (green) with native Zn-MCR-1-S (PDB ID: 6LI4) (orange) (RMSD value of 0.476 Å). **c,d,** Superimposition of **(c)** overall structures and **(d)** active sites of Au-MCR-1-S (green) with apo-MCR-1-S (PDB ID: 6LI5) (grey) (RMSD value of 0.573 Å). Au(I) ions are shown as orange spheres. Structural alignment was done over C_{α} residues using DaliLite and the images were generated using PyMOL.

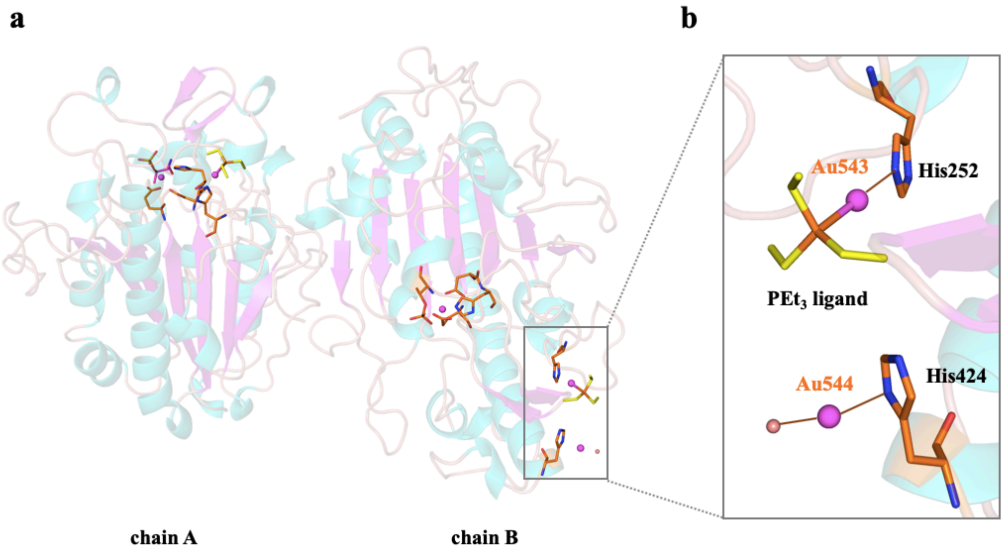

Supplementary Fig. 10 | a, Overall structure of Au-MCR-1-S. **b,** Binding of Au(I) ions (Au^{543} and Au^{544}) to the non-active sites in Au-MCR-1-S structure. Note that Au^{543} was only observed in chain B in the asymmetric unit of Au-MCR-1-S.

Supplementary Table 1 | Collection of X-ray crystallography data and refinement statistics for Au-NDM-1.

**Data collection**	**Au-NDM-1**
Wavelength (Å)	1.04005
Resolution range (Å)	36.88 - 1.21 (1.25 - 1.21)*
Space group	$P\ 2_1\ 2_1\ 2_1$
a, b, c (Å)	70.47, 73.76, 77.72
α, β, γ (º)	90.00, 90.00, 90.00
Unique reflections	124364 (12191)
Completeness (%)	99.50 (98.56)
Mean I/sigma(I)	23.55 (3.52)
Wilson B-factor	15.49
R_{merge}	0.0265 (0.236)
R_{means}	0.0375 (0.334)
Reflections for refinement	124343 (12189)
R_{work}	0.173 (0.265)
R_{free}	0.187 (0.274)
Number of non-hydrogen atoms	3766
RMS (bonds) (Å)	0.005
RMS (angles) (º)	1.18
Ramachandran favoured (%)	99.09
Ramachandran allowed (%)	0.91
Ramachandran outliers (%)	0.00

*Statistics for the highest-resolution shell are shown in parentheses.

Supplementary Table 2 | Bond length (Å) of Au(I) to coordinated ligands in Au-NDM-1.

Metal-ligand Distances (Å)	Au-NDM-1*		
	Au281	Au282	Au283
Glu152-Oε2	2.3		
Asp223-Oε2	2.2		
Glu227-Oε2	2.4		
HOH391-O	2.2		
Cys208-Sγ		2.5	
His250-Nε2		2.3	
Asp124-O		2.2	
HOH291-O		2.4	
His122-Nδ1			2.0
His120-Nε2			2.5
His189-Nε2			2.0
HOH410-O			1.9

*Values in parentheses are for the highest resolution shell.

Supplementary Table 3 | Susceptibility of MBL-positive or -negative strains against MER and AUR combination.

Strain	MER MIC (μg·mL^{-1}) in the presence of AUR at concentration stated (μg·mL^{-1})				FIC index*
	0	2	4	16	
E. coli BL21	0.03	0.03	0.015	0.015	0.625
NDM-HK	16	8	4	0.25	0.156
E. coli CKE	32	32	16	2	0.313
K. pneumoniae (NDM-1^{+})	64	32	8	2	0.156
E. coli (VIM-2^{+})	4	0.5	0.25	0.125	0.140
E. coli (IMP-4^{+})	8	2	1	0.0625	0.133
E. coli (CphA^{+})	4	1	1	0.5	0.375

*AUR was ineffective towards all the tested strains. MIC_{AUR}= 64 μg·mL^{-1} was used for the calculation of FICI.

**Supplementary Table 4 | ** Data collection and refinement statistics for apo-MCR-1-S, Au-MCR-1-S and Zn-MCR-1-S.

**Data collection**	**Apo-MCR-1-S**	**Zn-MCR-1-S**	**Au-MCR-1-S**
Space group	P1 21 1	P1 21 1	P1 21 1
a, b, c (Å)	47.16, 84.05,81.42	46.97, 84.20, 81.40	47.02, 84.49, 81.93
α, β, γ(°)	90.00, 98.51, 90.00	90.00, 98.78, 90.00	90.00, 98.64, 90.00
Wavelength (Å)	0.97915	0.97915	0.97915
Resolution (Å)	30.71~1.82(1.86~1.82)*	32.99~1.83(1.90~1.83*	43.22~1.60(1.66~1.60*
Multiplicity	2.0 (2.0)	2.0 (2.0)	2.0 (2.0)
Completeness (%)	96.73	98.87	99.83
Wilson B-factor	39.24	26.30	25.27
Reflections used in R_{free}	2482(263)	2570(278)	4252(392)
R_{work}/R_{free}	0.202/0.241	0.188/0.226	0.2635/0.2794
Macromolecular	5122	5027	5080
Protein residues	646	646	646
RMSD (bonds) (Å)	0.007	0.005	0.007
RMSD (angles) (°)	0.87	1.04	0.88
Ramachandran favoured (%)	96.54	97.51	97.01
Ramachandran allowed (%)	3.14	2.18	2.67
Ramachandran outliers (%)	0.31	0.31	0.31
Average B, all atoms ($Å^2$)	50.56	39.45	36.70
Water	75	329	176

*Values in parentheses are for the highest resolution shell.

Supplementary Table 5 Bond length (Å) of Zn(II) and Au(I) to coordinated in Zn-MCR-1-S and Au-MCR-1-S.

**Metal-ligand Distances (Å)**	**Zn-MCR-1-S***	**Au-MCR-1-S***		
	Zn542	Au542	Au543	Au544#
Thr285-Oγ	2.0	2.2		
His466-Nε2	2.1	2.2		
Asp465-Oδ1	2.0	2.2		
Glu246-Oε1	2.0	2.3		
His255-Nε2			2.2	
PEt_3-P1			2.5	
His424-Nδ1				2.2
HOH549-O				2.2

*Values in parentheses are for the highest resolution shell.
#Au544 was only seen in one chain in an asymmetric unit as shown in **Supplementary Fig. 10**.

Supplementary Table 6 | Susceptibility of MCR-positive or -negative strains against COL and AUR combination.

Strain	COL MIC ($\mu g \cdot mL^{-1}$) in the presence of AUR at concentration stated ($\mu g \cdot mL^{-1}$)				FIC index*
	0	0.625	1.25	5	
E. coli J53 (vector control)	0.5	0.25	0.25	0.25	0.531
MCR-1-J53	8	0.5	0.5	0.125	0.094
E. coli CKE	8	4	2	1	0.281
S. flexneri (MCR-1$^+$)	4	1	1	0.5	0.281
S. typhimurium (MCR-1$^+$)	16	2	1	0.25	0.125
K. pneumoniae (MCR-1$^+$)	64	16	8	2	0.125
E. asburiae (MCR-1$^+$)	8	1	0.5	0.125	0.125
E. aerogenes (MCR-1$^+$)	8	4	1	0.125	0.187
E. kobei (MCR-1$^+$)	8	0.5	0.25	0.125	0.094
MCR-1.5-J53	8	0.5	0.5	0.25	0.094
MCR-1.6-J53	8	0.5	0.5	0.25	0.094
MCR-1.7-J53	8	0.5	0.5	0.25	0.094
MCR-1.9-J53	8	0.5	0.5	0.25	0.094
MCR-1.10-J53	8	0.5	0.5	0.25	0.094
MCR-2.1-J53	8	0.5	0.5	0.25	0.094
MCR-3.1-J53	8	1	1	0.125	0.156
MCR-4.1-J53	8	1	0.5	0.25	0.125
MCR-5.1-J53	8	0.5	0.5	0.25	0.094
MCR-6.1-J53	8	0.5	0.5	0.25	0.094
MCR-7.1-J53	8	0.5	0.5	0.125	0.094

*AUR was ineffective towards all the tested strains. MIC_{AUR}= 40 $\mu g \cdot mL^{-1}$ was used for the calculation of FICI.

Supplementary Table 7 | Related information of bacterial strains.

Strain name	Relevant Genotype/Phenotype	Source/Reference
E. coli DH5a	Engineering strain	In house
E. coli BL21(DE3)	Engineering strain	In house
E. coli J53	Engineering strain	In house
E. coli CKE	Clinical isolate of *E. coli* CKE 1493; MCR-1$^+$, NDM-5$^+$; COL^R; MER^R	In house
NDM-HK	Clinical isolate of *E. coli* NDM-HK; NDM-1$^+$; MER^R	In house
NDM-BL21	*E. coli* BL21(DE3)(pET-28a-NDM-1); NDM-1$^+$; MER^R	In house
NDM-Rosetta	Rosetta(DE3)(pET-28a-NDM-1); NDM-1$^+$; MER^R	In house
C208A-BL21	*E. coli* BL21 (DE3)(pET-28a-NDM-1-C208A); NDM-1-C208A$^+$	In house
MCR-1-BL21	*E. coli* BL21(DE3)(pET-28a-MCR-1); MCR-1$^+$; COL^R	This study
MCR-1-S-BL21	*E. coli* BL21(DE3)(pET-15b-MCR-1-S); MCR-1-S$^+$;	This study
E. coli (VIM-2$^+$)	*E. coli* BL21(DE3)(pET-28a-VIM-2); VIM-2$^+$; MER^R	In house
E. coli (IMP-4$^+$)	*E. coli* BL21(DE3)(pET-28a-IMP-4); IMP-4$^+$; MER^R	In house
E. coli (CphA$^+$)	*E. coli* BL21(DE3)(pET-28a-CphA); CphA$^+$; MER^R	This study
K. pneumoniae (NDM-1$^+$)	Clinical isolate of *Klebsiella pneumonia*; NDM-1$^+$; MER^R	Patrick CY WOO
MCR-1-J53	*E. coli* J53 (pCR-XL-TOPO-MCR-1); MCR-1$^+$; COL^R	This study
MCR-1.5-J53	*E. coli* J53 (pET-28a-MCR-1.5); MCR-1.5$^+$; COL^R	This study
MCR-1.6-J53	*E. coli* J53 (pET-28a-MCR-1.6); MCR-1.6$^+$; COL^R	This study
MCR-1.7-J53	*E. coli* J53 (pET-28a-MCR-1.7); MCR-1.7$^+$; COL^R	This study
MCR-1.9-J53	*E. coli* J53 (pET-28a-MCR-1.9); MCR-1.9$^+$; COL^R	This study
MCR-1.10-J53	*E. coli* J53 (pET-28a-MCR-1.10); MCR-1.10$^+$; COL^R	This study
MCR-2.1-J53	*E. coli* J53 (pET-28a-MCR-2.1); MCR-2.1$^+$; COL^R	This study
MCR-3.1-J53	*E. coli* J53 (pET-28a-MCR-3.1); MCR-3.1$^+$; COL^R	This study
MCR-4.1-J53	*E. coli* J53 (pET-28a-MCR-4.1); MCR-4.1$^+$; COL^R	This study
MCR-5.1-J53	*E. coli* J53 (pET-28a-MCR-5.1); MCR-5.1$^+$; COL^R	This study
MCR-6.1-J53	*E. coli* J53 (pET-28a-MCR-6.1); MCR-6.1$^+$; COL^R	This study
MCR-7.1-J53	*E. coli* J53 (pET-28a-MCR-7.1); MCR-7.1$^+$; COL^R	This study
S. flexneri (MCR-1$^-$)	Clinical isolate of *Shigella flexneri*	In house
S. flexneri (MCR-1$^+$)	Clinical isolate of *Shigella flexneri* CH27; MCR-1$^+$; COL^R	In house
S. typhimurium (MCR-1$^+$)	Clinical isolate of *Salmonella typhimurium* 0839; MCR-1$^+$; COL^R	In house
K. pneumoniae (MCR-1$^+$)	Clinical isolate of *K. pneumoniae* 9607; MCR-1$^+$; COL^R	In house
E. asburiae (MCR-1$^+$)	Clinical isolate of *Enterobacter asburiae* 2658; MCR-1$^+$; COL^R	In house
E. aerogenes (MCR-1$^+$)	Clinical isolate of *Enterobacter aerogenes* 7014; MCR-1$^+$; COL^R	In house
E. kobei (MCR-1$^+$)	Clinical isolate of *Enterobacter kobei* 4113; MCR-1$^+$; COL^R	In house

Supplementary Table 8 | Summary of primer sequences (5'-3').

Primer name	
MCR-1.1 F	**5'-** TCAGAGCTCATGATGCAGCATACTTCTG **-3'**
MCR-1.1 R	**5'-** TCAGGATCCTCAGCGGATGAATGCG **-3'**
MCR-1.5 F	**5'-** TCACCATGGGGATGATGCAGCATACTTCTG **-3'**
MCR-1.5 R	**5'-** TCACTCGAGTCAGCGGATGAATGCG **-3'**
MCR-1.6 F	**5'-** TCACCATGGGGATGATGCAGCATACTTCTG **-3'**
MCR-1.6 R	**5'-** TCACTCGAGTCAGCGGATGAATGCG **-3'**
MCR-1.7 F	**5'-** TCACCATGGGGATGATGCAGCATACTTCTG **-3'**
MCR-1.7 R	**5'-** TCACTCGAGTCAGCGGATGAATGCG **-3'**
MCR-1.9 F	**5'-** TCACCATGGGGATGATGCAGCATACTTCTG **-3'**
MCR-1.9 R	**5'-** TCACTCGAGTCAGCGGATGAATGCG **-3'**
MCR-1.10 F	**5'-** TCACCATGGGGATGATGCAGCATACTTCTG **-3'**
MCR-1.10 R	**5'-** TCACTCGAGTCAGCGGATGAATGCG **-3'**
MCR-2.1 F	**5'-** TCACCATGGGGATGACATCACATCAC **-3'**
MCR-2.1 R	**5'-** TCACTCGAGTTACTGGATAAATGCCG **-3'**
MCR-3.1 F	**5'-** TCACCATGGGGATGCCTTCCCTTATAAAAAT **-3'**
MCR-3.1 R	**5'-** TCACTCGAGTTATTGAACATTACGACATTG **-3'**
MCR-4.1 F	**5'-** TCACCATGGGGATGATTTCTAGATTTAAG **-3'**
MCR-4.1 R	**5'-** TCACTCGAGTAATACCTGCAAGGTGC **-3'**
MCR-5.1 F	**5'-** TCACCATGGGGATGCGGTTGTCTGCATTTATC **-3'**
MCR-5.1 R	**5'-** TCACTCGAGTCATTGTGGTTGTCCTTTTC **-3'**
MCR-6.1 F	**5'-** TCACCATGGGGATGACACAGCATAGTCC **-3'**
MCR-6.1 R	**5'-** TCAGGATCCTCAGCGGATGAATGCG **-3'**
MCR-7.1 F	**5'-** TCACCATGGGGATGCGCATCACGCTCGG**-3'**
MCR-7.1 R	**5'-** TCACTCGAGTTACTGCACGGTGCGGC **-3'**
MCR-1-full length F	**5'-** TCACATATGATGCAGCATACTTCTGTGTG**-3'**
MCR-1-full length R	**5'-** TCAGGATCCTCAGCGGATGAATGCGGTGCGGTC **-3'**
pET15b mutation F	**5'-** GATATACCTTGGGCAGCAGCCATC**-3'**
pET15b mutation R	**5'-** CTGCCCAAGGTATATCTCCTTC**-3'**

*These primers involved in the experiment. The restriction endonucleases digesting sites and mutated nucleotides are highlighted in blue and red color, respectively. Nonsense refinements were performed on NcoI sites of *mcr-3*, *mcr-4*, *mcr-5* and *mcr-7* genes to avoid the digestion by corresponding enzyme.
