# Supplementary material for: Resensitizing carbapenem- and colistin-resistant bacteria to antibiotics using auranofin
Source: Nat Commun. 2020 Oct 16;11:5263. doi: 10.1038/s41467-020-18939-y (PMC7568570; doi:10.1038/s41467-020-18939-y)
Supplement: Supplementary file 3 — Description of Additional Supplementary Files [file 41467_2020_18939_MOESM3_ESM.docx]

**Description of Additional Supplementary Files**

File Name: Supplementary Data 1

Description: Full scan of western blot for panels of Figures 1e; 3e.

File Name: Supplementary Data 2

Description: Full sequences of plasmid as described in ‘Construction of plasmids’ in Methods section.
