# Supplementary material for: Resensitizing carbapenem- and colistin-resistant bacteria to antibiotics using auranofin
Source: Nat Commun. 2020 Oct 16;11:5263. doi: 10.1038/s41467-020-18939-y (PMC7568570; doi:10.1038/s41467-020-18939-y)

Full scan of western blot for panels of figures 1e; 3e

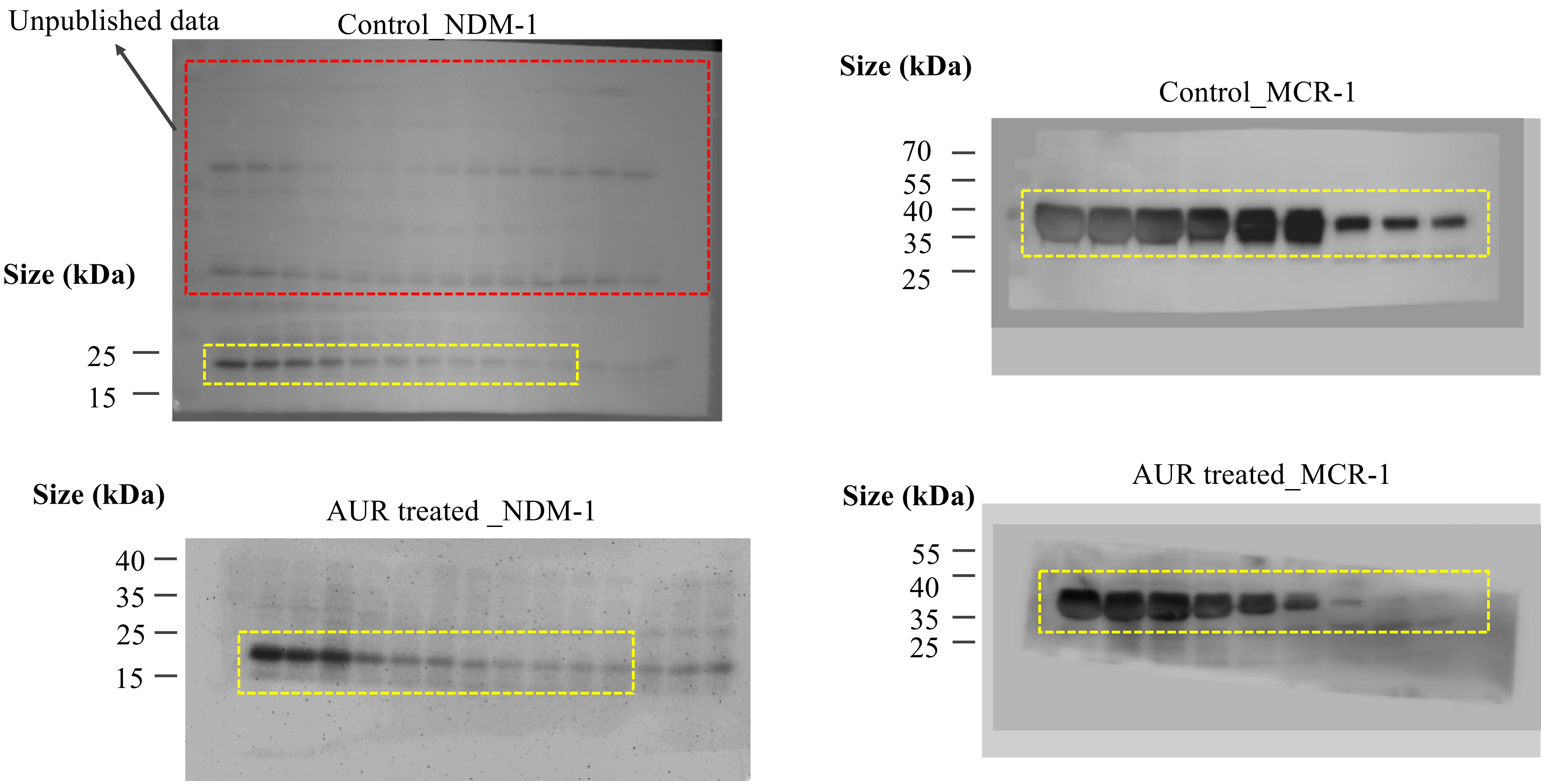

Supplement: Supplementary file 5 — Supplementary Data 2 [file 41467_2020_18939_MOESM5_ESM.pdf]
