# Supplementary material for: Resensitizing carbapenem- and colistin-resistant bacteria to antibiotics using auranofin
Source: Nat Commun. 2020 Oct 16;11:5263. doi: 10.1038/s41467-020-18939-y (PMC7568570; doi:10.1038/s41467-020-18939-y)
Supplement: Supplementary file 6 — Reporting Summary [file 41467_2020_18939_MOESM6_ESM.pdf]

nature research

Corresponding author(s): Hongzhe Sun

Last updated by author(s): Sep 11, 2020

Reporting Summary

Nature Research wishes to improve the reproducibility of the work that we publish. This form provides structure for consistency and transparency in reporting. For further information on Nature Research policies, see Authors & Referees and the Editorial Policy Checklist .

Statistics

For all statistical analyses, confirm that the following items are present in the figure legend, table legend, main text, or Methods section.

n/a	Confirmed	
☐	☒	The exact sample size (n) for each experimental group/condition, given as a discrete number and unit of measurement
☐	☒	A statement on whether measurements were taken from distinct samples or whether the same sample was measured repeatedly
☐	☒	The statistical test(s) used AND whether they are one- or two-sided<br>*Only common tests should be described solely by name; describe more complex techniques in the Methods section.*
☒	☐	A description of all covariates tested
☒	☐	A description of any assumptions or corrections, such as tests of normality and adjustment for multiple comparisons
☐	☒	A full description of the statistical parameters including central tendency (e.g. means) or other basic estimates (e.g. regression coefficient) AND variation (e.g. standard deviation) or associated estimates of uncertainty (e.g. confidence intervals)
☐	☒	For null hypothesis testing, the test statistic (e.g. F, t, r) with confidence intervals, effect sizes, degrees of freedom and P value noted<br>*Give P values as exact values whenever suitable.*
☒	☐	For Bayesian analysis, information on the choice of priors and Markov chain Monte Carlo settings
☒	☐	For hierarchical and complex designs, identification of the appropriate level for tests and full reporting of outcomes
☒	☐	Estimates of effect sizes (e.g. Cohen's d, Pearson's r), indicating how they were calculated

Our web collection on statistics for biologists contains articles on many of the points above.

Software and code

Policy information about availability of computer code

Data collection	Crystallography data were collected by HKL2000 (version 716). CCD detector was ADSC QUANTUM 315r and Radaition source was SSRF beamline BL17U.
Data analysis	MS data was processed using FlexAnalysis (version 1.2, Bruker Daltonics).The diffraction data were reduced with XDS54. The Phaser from CCP4 suite (7.0.078) and Phenix (version 1.15-3459) were used for data refinement and finalization. Cycles of refinement with the anomalous data and with careful manual rebuilding were done by using Refmac 5.8.0135 and Coot version 6, respectively. TLS refinement was used in the later stages of data processing. The final models were analyzed with MolProbity version 4.4. Structural alignment was done over Cα residues using DaliLite version 5. All of the structural illustrations were generated using the software PyMOL1.8.0.0. Image J (1.52a) was used to quantify the signals of each band for cellular thermal shift analysis. All other analyses were carried out using GraphPad Prism software 8.0 (GraphPad Software, Inc., La Jolla, CA)

For manuscripts utilizing custom algorithms or software that are central to the research but not yet described in published literature, software must be made available to editors/reviewers. We strongly encourage code deposition in a community repository (e.g. GitHub). See the Nature Research guidelines for submitting code & software for further information.

Data

Policy information about availability of data

All manuscripts must include a data availability statement. This statement should provide the following information, where applicable:

- Accession codes, unique identifiers, or web links for publicly available datasets
- A list of figures that have associated raw data
- A description of any restrictions on data availability

All data are available in the main manuscript or in the Supplementary Information, except that the structure factors for Au-NDM-1, Zn-NDM-1, Au-MCR-1-S, Zn-MCR-1-S, and apo-MCR-1-S are deposited at Protein Data Bank with accessing codes of 6LHE, 5ZGE, 6LI6, 6LI4 and 6LI5, respectively.

Field-specific reporting

Please select the one below that is the best fit for your research. If you are not sure, read the appropriate sections before making your selection.

- [x] Life sciences
- [ ] Behavioural & social sciences
- [ ] Ecological, evolutionary & environmental sciences

For a reference copy of the document with all sections, see nature.com/documents/nr-reporting-summary-flat.pdf

Life sciences study design

All studies must disclose on these points even when the disclosure is negative.

Sample size	The sample size and n/group was chosen based on similar experiments conducted by previous studies (refs 31, 34 and 43) using the same or similar mouse strains. The sample size of n= 4 each group was chosen for CFU counting in animal tissue and n= 6 for animal survival studies. For in vitro biochemical studies, three biological repeats, followed by repeating the experiments twice for confirmation were widely accepted and used in published papers.
Data exclusions	No data were excluded from the analysis.
Replication	All the experiments were subjected to three biological replicates unless specified. All attempts at replication were successful.
Randomization	All the animals were numbered and allocated into groups using a simple randomization of excel-generated random numbers. To avoid biases, we also assured that different treatments were performed on the same day. All the animals were randomized to cages for each experiment and had free access to food and water.
Blinding	Mice were blindly allocated to each group. The sample classification were replaced by simple marks during data collection and analysis. The investigators were not blinded to the allocation during experiments and outcome assessment. Data collection and analysis were performed by the same people.

Reporting for specific materials, systems and methods

We require information from authors about some types of materials, experimental systems and methods used in many studies. Here, indicate whether each material, system or method listed is relevant to your study. If you are not sure if a list item applies to your research, read the appropriate section before selecting a response.

Materials & experimental systems

n/a	Involved in the study	
[ ]	[x]	Antibodies
[x]	[ ]	Eukaryotic cell lines
[x]	[ ]	Palaeontology
[ ]	[x]	Animals and other organisms
[x]	[ ]	Human research participants
[x]	[ ]	Clinical data

Methods

n/a	Involved in the study	
[x]	[ ]	ChIP-seq
[x]	[ ]	Flow cytometry
[x]	[ ]	MRI-based neuroimaging

Antibodies

Antibodies used	NDM-1 polyclonal, antibody with 1:1000 dilution (NBP1-77688, NOVUS Biologicals), MCR-1 polyclonal antibody with 1:1000 dilution (CSB-PA745804LA01ENL, Cusabio Technology LLC), Anti-rabbit IgG, HRP-linked Antibody with 1:3000 dilution, #7074, Cell Signaling Technology, Inc.
Validation	All the antibodies are validated for the indicated use by the manufacturers.<br>For NDM-1 polyclonal, antibody (NBP1-77688, NOVUS Biologicals),<br>Species: Bactreia, E. coli, Application:WB, Clonality: Polyclonal, Host: Rabbit, Conjugate: Unconjugated<br>https://www.novusbio.com/products/ndm-1-antibody_nbp1-77688#supportresearch<br><br>For MCR-1 polyclonal antibody (CSB-PA745804LA01ENL, Cusabio Technology LLC)<br>Species: Escherichia coli, Applications: ELISA, Clonality: Polyclonal, Host: Rabbit, Conjugate: Unconjugated<br>https://www.cusabio.com/Polyclonal-Antibody/mcr1-Antibody-11910132.html<br><br>For Anti-rabbit IgG, HRP-linked Antibody<br>Source/Isotype: Goat, Applications: WB<br>https://media.cellsignal.com/pdf/7074.pdf

Animals and other organisms

Policy information about studies involving animals; ARRIVE guidelines recommended for reporting animal research

Laboratory animals	Six to eight weeks old, female, 18~22 g of weight Balb/c mice were purchased from Charles River Laboratories, Inc. All the animals were randomized to cages for each experiment and had free access to food and water. Those animals are generally maintained at 25 °C and 40% humidity.
Wild animals	This study did not involve any wild animals.
Field-collected samples	This study did not involve samples collected from the field.
Ethics oversight	All experiments were approved by, and performed in accordance with the guidelines approved by Committee on the Use of Live Animals in Teaching and Research (CULATR), The University of Hong Kong (Reference code: CULATR 4008-16).

Note that full information on the approval of the study protocol must also be provided in the manuscript.
